# Supplementary material for: Target Identification of 22-(4-Pyridinecarbonyl) Jorunnamycin A, a Tetrahydroisoquinoline Derivative from the Sponge Xestospongia sp., in Mediating Non-Small-Cell Lung Cancer Cell Apoptosis
Source: Molecules. 2022 Dec 15;27(24):8948. doi: 10.3390/molecules27248948 (PMC9782168; doi:10.3390/molecules27248948)
Supplement: Supplementary file 1 [file molecules-27-08948-s001.zip › molecules-2066419-supplementary.pdf]

Supplementary information for

Target identification of 22-(4-pyridinecarbonyl) jorunnamycin A, a tetrahydroisoquinoline derivative from the sponge *Xestospongia* sp., in mediating non-small cell lung cancer cell apoptosis

Iksen Iksen[1], Suwimon Sinsook[2,3], Onsurang Wattanathamsan[1], Koonchira Buaban[3,4], Supakarn Chamni[3,4], Varisa Pongrakhananon[1, 5 *]

[1] Department of Pharmacology and Physiology, Faculty of Pharmaceutical Sciences, Chulalongkorn University, Bangkok, 10330 Thailand

[2] Pharmaceutical Sciences and Technology Program, Faculty of Pharmaceutical Sciences, Chulalongkorn University, Bangkok 10330, Thailand

[3] Department of Pharmacognosy and Pharmaceutical Botany, Faculty of Pharmaceutical Sciences, Chulalongkorn University, Bangkok 10330, Thailand

[4] Natural Products and Nanoparticles Research Unit (NP2), Chulalongkorn University, Bangkok 10330, Thailand

[5] Preclinical Toxicity and Efficacy, Assessment of Medicines and Chemicals Research Unit, Chulalongkorn University, Bangkok, 10330 Thailand

***Corresponding author**

Varisa Pongrakhananon, Department of Pharmacology and Physiology,

Faculty of Pharmaceutical Sciences, Chulalongkorn University, 254 Phayathai, Wangmai, Pathumwan, Bangkok, Thailand 10330

Tel: +662-218-8325; Fax: +662-218-8340

Email: Varisa.p@pharm.chula.ac.th

1. Supplementary Materials and Methods

1.1 Cell culture

Lung epithelial BEAS-2B cells were obtained from the American Type Culture Collection (ATCC, Virginia, USA). Cells were cultured in Dulbecco's modified Eagle's medium (DMEM). All media were supplemented with 10% fetal bovine serum (FBS), 100 U/mL penicillin–streptomycin antibiotic solution and 2 mM L-glutamine. Cells were maintained in a 37 °C humidified incubator with 5% CO_2.

2. Supplementary Figures and Tables

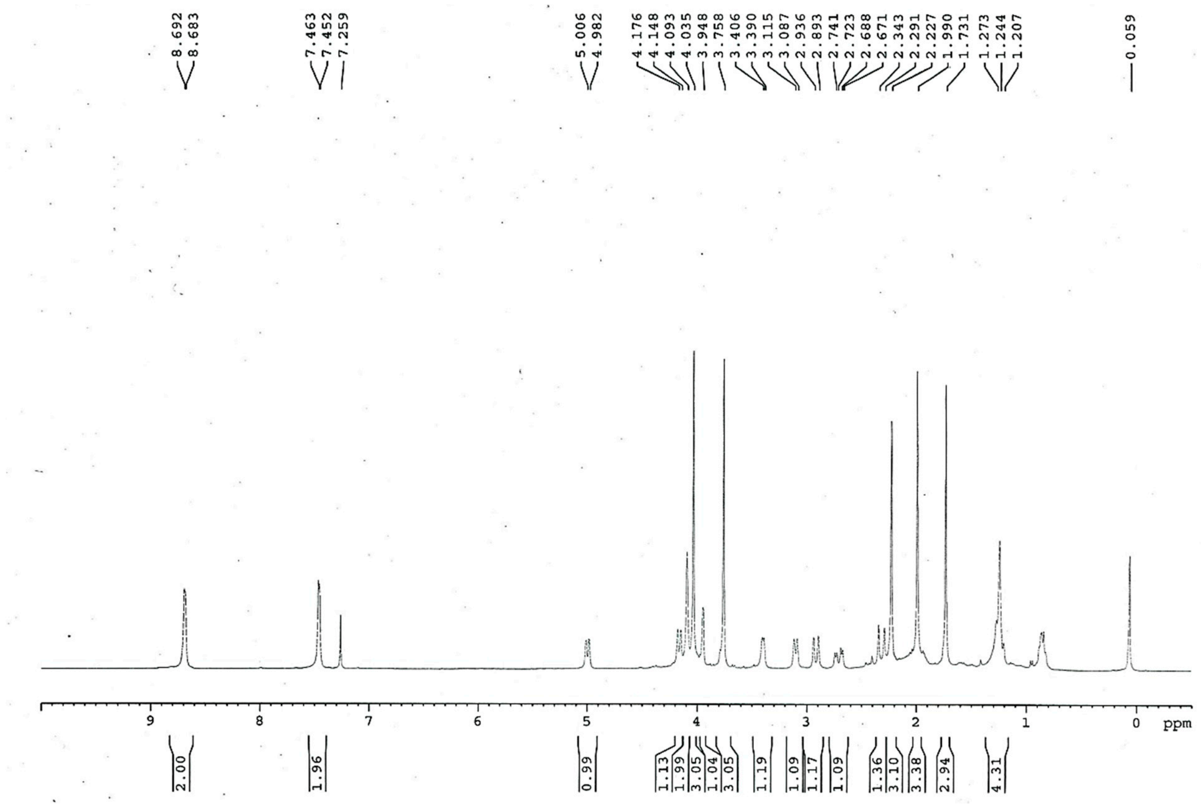

Figure S1. ^{1}H NMR (400 MHz) spectrum of 22-(4'py)-JA (12 mg) in $CDCl_3$. ^{1}H-NMR ($CDCl_3$, 400 MHz) d in ppm: 8.68 (2H, d, J = 3.6 Hz, 2'-H, 6'-H), 7.47 (2H, d, J = 4.4 Hz, 3'-H, 5'-H), 4.99 (1H, d, J = 9.6 Hz, 22-Ha), 4.16 (1H, d, J = 11.2 Hz, 22-Hb), 4.09 (1H, s, 21-H,1-H), 4.04 (3H, s, 7-OCH_3), 3.95 (1H, s, 11-H), 3.76 (3H, s, 17-OCH_3), 3.40 (1H, d, J = 6.4 Hz, 13-H), 3.10 (1H, d, J = 11.2 Hz, 3-H), 2.92 (1H, d, J = 17.2 Hz, 4-Ha), 2.71 (1H, dd, J = 21.2, 7.2 Hz, 14-Ha), 2.34 (1H, d, J = 20.8 Hz, 14-Hb), 2.23 (3H, s, NCH_3), 1.99 (3H, s, 6-CH_3), 1.73 (3H, s, 16-CH_3), 1.24 (1H, overlapped, 4-Hb)

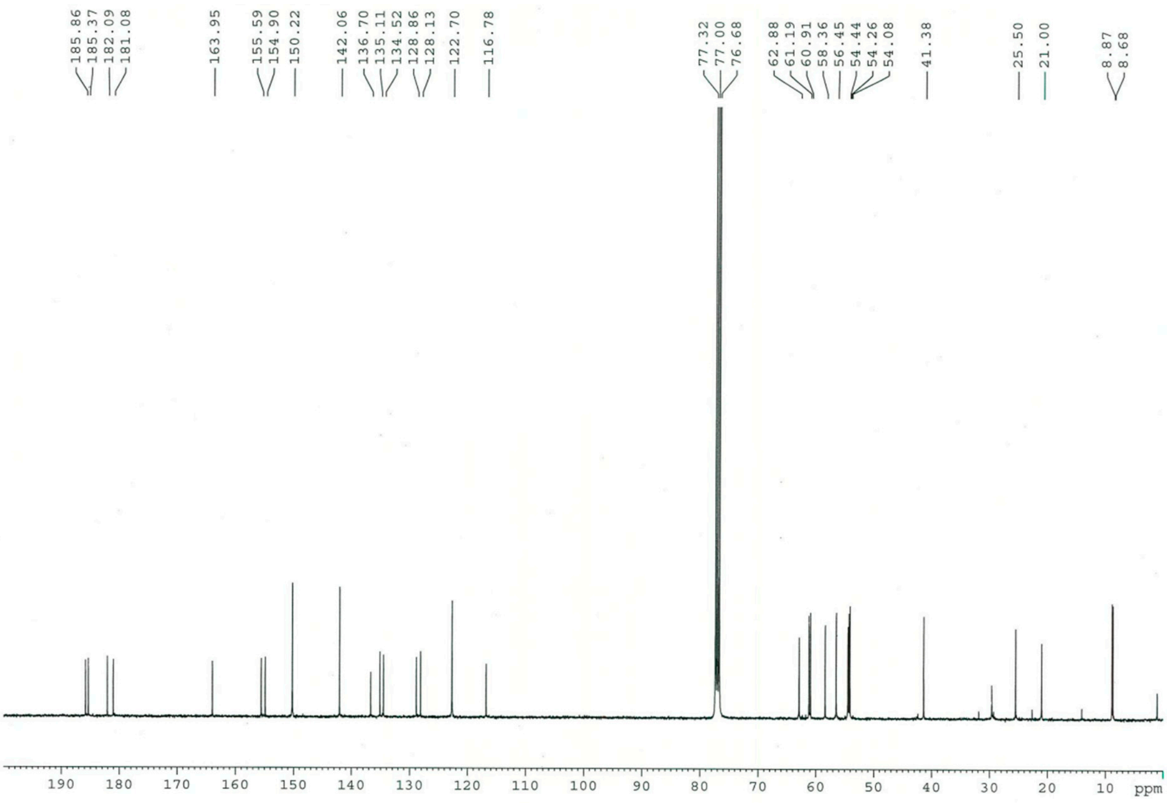

Figure S2. ^{13}C NMR (100 MHz) spectrum of 22-(4′py)-JA (12 mg) in $CDCl_3$. ^{13}C-NMR ($CDCl_3$, 100 MHz) d in ppm: ^{13}C-NMR ($CDCl_3$, 100 MHz) d in ppm: 185.9 (C-15), 185.4 (C-5), 182.1 (C-18), 181.1 (C-8), 163.9 (C-24), 155.6 (C-7), 154.9 (C-17), 150.2 (C-2C, C-6C), 142.1 (C-10), 142.1 (C-20), 136.7 (C-1C), 135.1 (C-9), 134.5 (C-19), 128.9 (C-6), 128.1 (C-16), 122.7 (C-3C, C-5C), 116.8 (21-CN), 62.9 (C-22), 61.2 (17-OCH_3), 60.9 (7-OCH_3), 58.4 (C-21), 56.5 (C-1), 54.4 (C-13), 54.3 (C-3), 54.1 (C-11), 41.4 (NCH_3), 25.5 (C-4), 21.0 (C-14), 8.9 (6-CH_3), 8.7 (16-CH_3).

A High resolution-ESI-MS

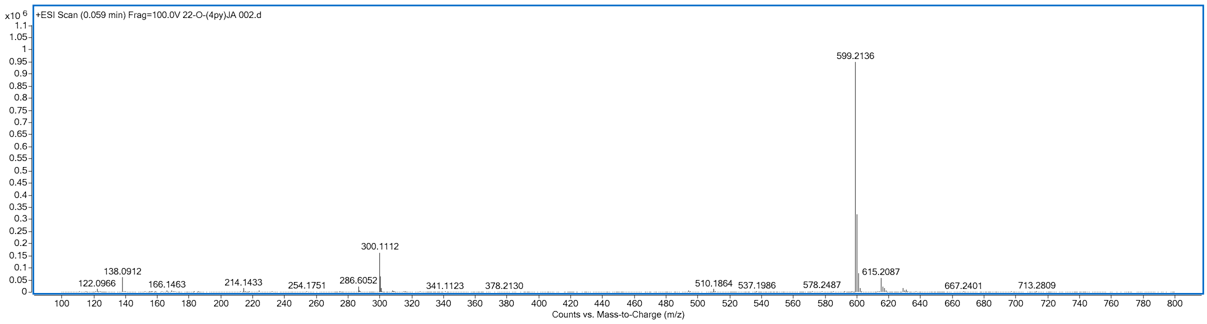

B MS-MS profile

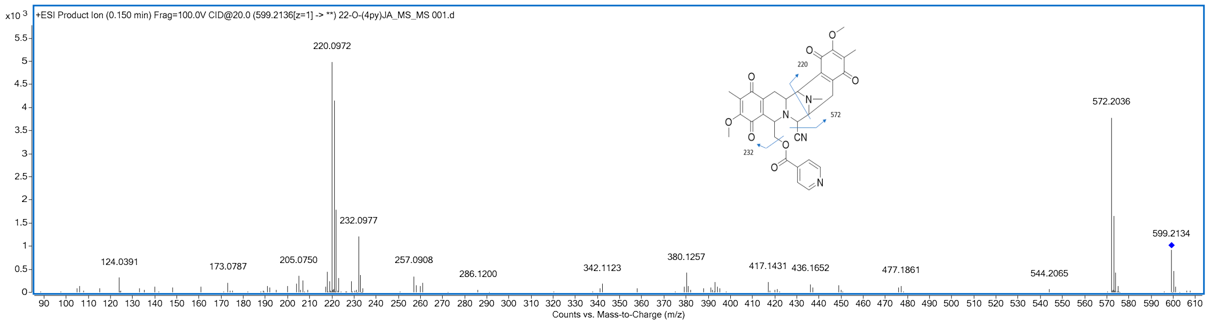

Figure S3 High resolution-ESI-MS of 22-(4′py)-JA; (A) HR-ESI-MS calculated for $C_{32}H_{31}N_4O_8$, m/z 599.2136 $[M+H]^+$, observed m/z 599.2136. (B) MS-MS profile found m/z 599.2134, 572.2036, 232.0977, 220.0972.

A Positive mode

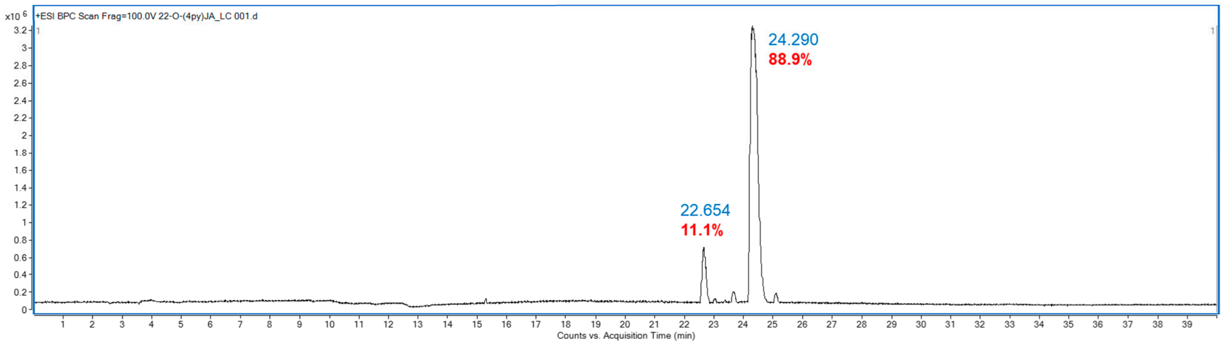

B Negative mode

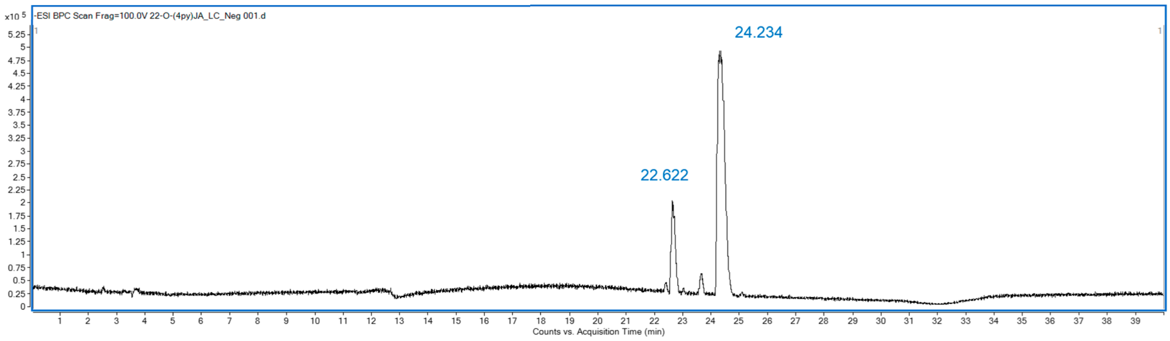

Figure S4. Purity analysis of 22-(4′py)-JA by LC-MS; (A) Positive mode. (B) Negative mode. Percent purity based on LC-MS positive mode is 88.9%. LC-MS condition: TOF/Q-TOF Mass Spectrometer G6540B Agilent, Gas Flow 10 L/min, Nebulizer 30 psig, Eject Speed 200.0 µL/min, Injection Volume 10.00 µL, Flow 0.500 mL/min, Solvent A 0.1% formic acid in H_2O. 0.1% formic acid in MeOH: Acetonitrile (1:1 v/v), 40 min.

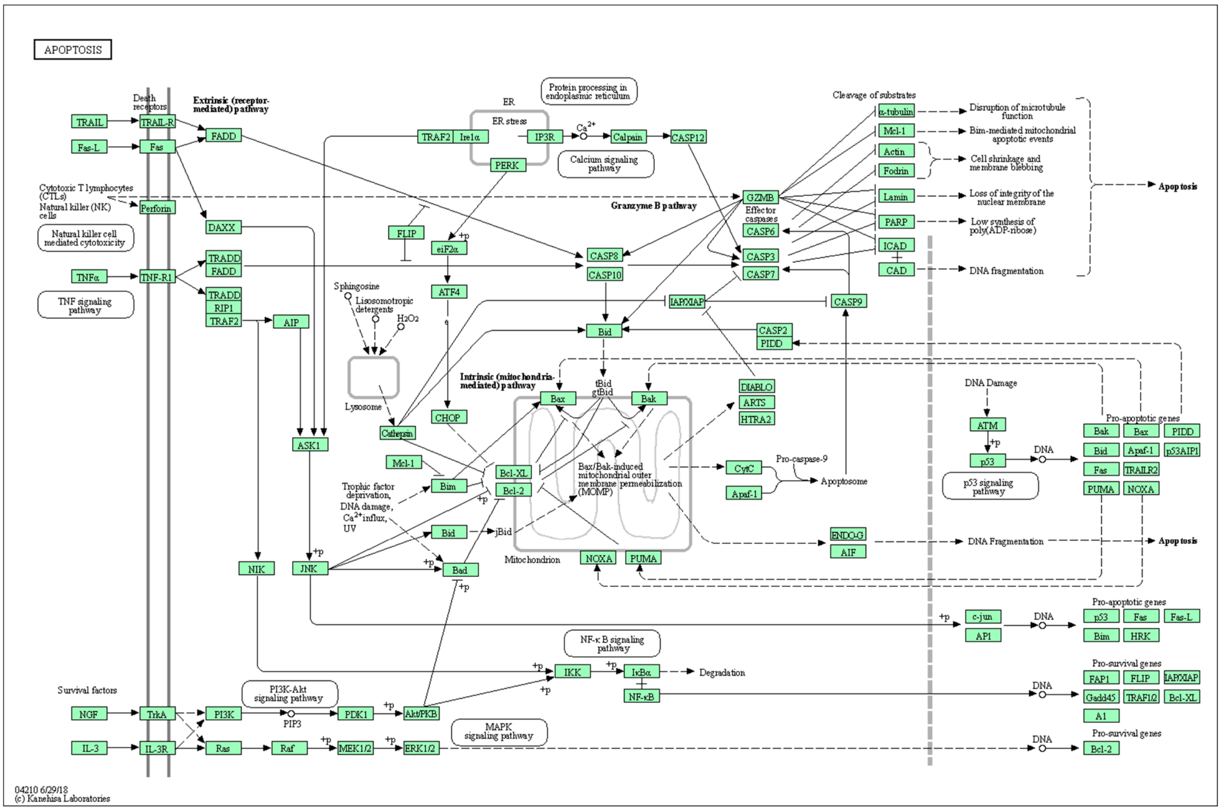

Figure S5. Apoptosis signaling pathway (hsa04210) constructed by KEGG database.

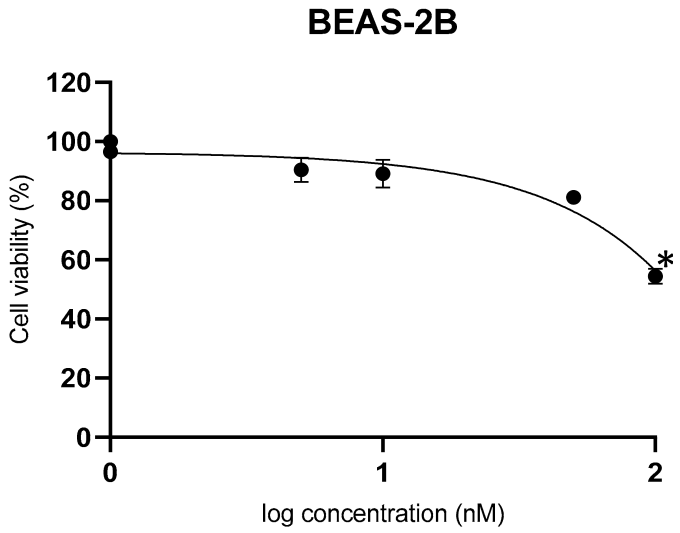

Figure S6. Cytotoxicity of 22-(4'py)-JA in normal lung epithelial BEAS-2B cells. BEAS-2B cells were treated with 22-(4'py)-JA for 48 h. Cytotoxic effects of 22-(4'py)-JA were evaluated by MTT assay and represented as a percentage of cell viability. Data are the mean ± SD (n = 3). *$p < 0.05$ vs. untreated control cells.

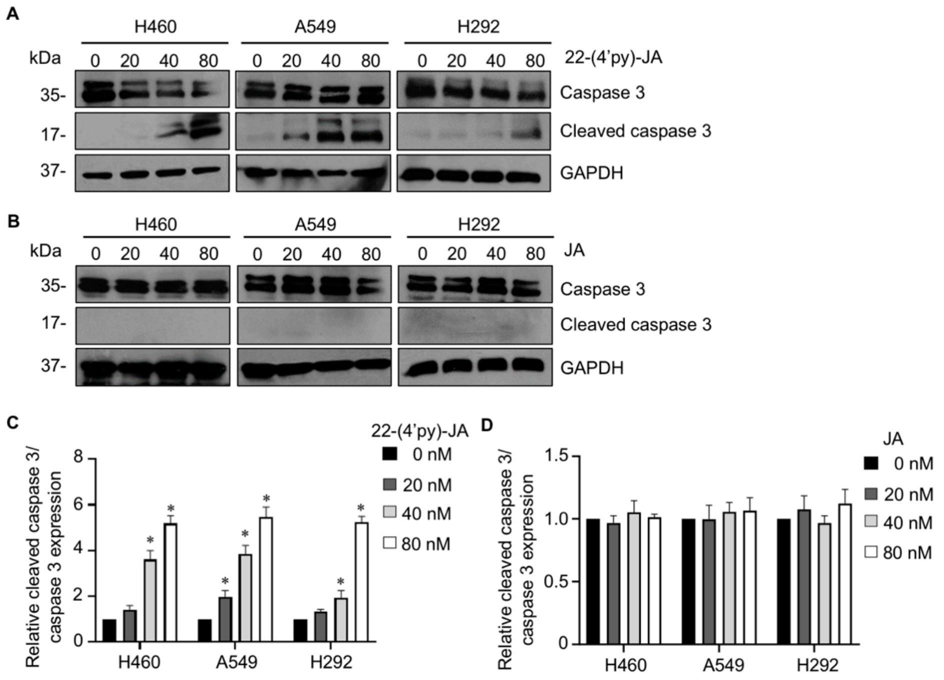

Figure S7. The effect of 22-(4'py)-JA and JA on caspase-3 activation. H460, A549, and H292 cells were incubated with either 22-(4'py)-JA (A) or JA (B) for 24 h. The expression levels of cleaved caspase-3 and total caspase-3 were analyzed by immunoblotting. Blots were reprobed with anti-GAPDH as a loading control. Representative blots from triplicate independent experiments are shown. Densitometry analyses of protein expression from the treatment of 22-(4'py)-JA (C) and JA (D) were performed and presented as a relative value to the control group. Data are the mean ± SD (n = 3). *$p < 0.05$ vs. untreated control cells.

Table S1. List of potential targets of 22-(4′py)-JA from SwissTargetPrediction.

ABCB1	CDK9	HCRTR2	MAPK3	PDE7A
ABCC1	CFD	HDAC1	MAPK8	PDPK1
ADAM17	CFTR	HDAC10	MAPK9	PFKFB3
ADORA1	CTSB	HDAC11	MLNR	PIK3CA
ADORA2A	CTSK	HDAC2	MMP13	PIK3CD
ADRA1A	CTSL	HDAC3	MMP2	PLA2G7
ADRA1B	CTSV	HDAC6	MMP3	PLAT
ADRA1D	DGAT1	HDAC8	MMP8	PRKDC
AGTR1	DHFR	HTR1A	MMP9	PTK2
AURKA	DHODH	IGF1R	MTOR	RPS6KA3
CAPN1	DRD4	JAK2	MYLK	S1PR3
CCKAR	EGFR	KDR	P2RX3	SCARB1
CCKBR	ELANE	KLKB1	PAK4	SLC33A1
CCND1	ERBB2	KNG1	PARP1	SLC6A4
CDK1	F2	LGMN	PDE10A	SRC
CDK2	F10	MAP2K1	PDE2A	SSTR3
CDK4	FNTA	MAP3K7	PDE4A	SYK
CDK5	GABRA5	MAPK1	PDE4B	TGM2
CDK5R1	GSK3B	MAPK10	PDE4D	TTK
CDK7	HCRTR1	MAPK14	PDE5A	TYMS

Table S2. Core targets of 22-(4′py)-JA against non-small cell lung cancer were ranked according to the degree values. The top 26 targets are highlighted in grey.

No	Name	Degree	Average Shortest Path Length	Betweenness Centrality	Closeness Centrality	Clustering Coefficient
1	SRC	44	1.460526316	0.190924974	0.684684685	0.287526427
2	MAPK3	37	1.565789474	0.094593765	0.638655462	0.334834835
3	EGFR	34	1.605263158	0.066548491	0.62295082	0.390374332
4	CCND1	33	1.631578947	0.048820227	0.612903226	0.450757576
5	MAPK1	33	1.592105263	0.093617362	0.628099174	0.380681818
6	MTOR	32	1.657894737	0.044310966	0.603174603	0.471774194
7	MMP9	31	1.671052632	0.067337048	0.598425197	0.397849462
8	ERBB2	28	1.697368421	0.028185505	0.589147287	0.494708995
9	MMP2	27	1.75	0.031940748	0.571428571	0.415954416
10	CDK4	25	1.776315789	0.033334762	0.562962963	0.45
11	MAP2K1	25	1.763157895	0.019332587	0.567164179	0.506666667
12	MAPK14	23	1.789473684	0.013198592	0.558823529	0.517786561
13	PIK3CA	23	1.828947368	0.0100228	0.54676259	0.56916996
14	PTK2	23	1.776315789	0.016695575	0.562962963	0.553359684
15	HDAC1	22	1.828947368	0.02580054	0.54676259	0.437229437
16	CDK2	21	1.855263158	0.028638091	0.539007092	0.428571429
17	GSK3B	21	1.828947368	0.012331244	0.54676259	0.514285714
18	MAPK8	20	1.842105263	0.00632414	0.542857143	0.636842105
19	PARP1	20	1.842105263	0.013122922	0.542857143	0.547368421
20	CDK1	20	1.907894737	0.021354067	0.524137931	0.478947368
21	JAK2	18	1.855263158	0.003380625	0.539007092	0.745098039
22	HDAC6	18	1.894736842	0.047889922	0.527777778	0.333333333
23	IGF1R	18	1.881578947	0.001286908	0.531468531	0.830065359
24	KDR	17	1.907894737	0.004594691	0.524137931	0.691176471
25	MMP3	15	1.934210526	0.022575186	0.517006803	0.485714286
26	F2	15	1.973684211	0.022480299	0.506666667	0.380952381
27	HDAC2	13	2.078947368	0.009649474	0.481012658	0.525641026
28	CTSB	13	2.065789474	0.011325768	0.484076433	0.371794872
29	CDK5	13	2.026315789	0.00512819	0.493506494	0.58974359
30	PRKDC	13	2.223684211	0.007280656	0.449704142	0.423076923
31	ABCB1	12	2.013157895	0.008941845	0.496732026	0.575757576
32	TYMS	12	2.263157895	0.018326529	0.441860465	0.5
33	KNG1	12	2.092105263	0.018132579	0.477987421	0.363636364
34	AURKA	12	2.171052632	0.00475996	0.460606061	0.575757576

35	DHFR	11	2.263157895	0.014802904	0.441860465	0.472727273
36	SYK	11	2.052631579	0.001335473	0.487179487	0.745454545
37	PIK3CD	11	2.131578947	0.000682	0.469135802	0.818181818
38	AGTR1	10	2.026315789	0.001601436	0.493506494	0.688888889
39	CTSL	10	2.236842105	0.002798608	0.447058824	0.511111111
40	RPS6KA3	10	2.039473684	0.000475	0.490322581	0.866666667
41	MAPK9	10	2.039473684	0.001607833	0.490322581	0.777777778
42	MAP3K7	10	2.184210526	0.001939282	0.457831325	0.577777778
43	MAPK10	9	2.157894737	0.000569	0.463414634	0.833333333
44	CAPN1	9	2.105263158	0.006371526	0.475	0.333333333
45	CDK9	9	2.302631579	0.000759	0.434285714	0.75
46	ELANE	9	2.368421053	0.00439699	0.422222222	0.5
47	PDPK1	9	2.184210526	0.001066589	0.457831325	0.666666667
48	ADAM17	8	2.092105263	0.000174	0.477987421	0.857142857
49	CDK5R1	8	2.355263158	0.001450666	0.424581006	0.571428571
50	CDK7	8	2.381578947	0.000189	0.419889503	0.857142857
51	PLAT	8	2.289473684	0.004174091	0.436781609	0.535714286
52	MMP13	7	2.315789474	0.001192613	0.431818182	0.523809524
53	CTSK	7	2.263157895	0.001102356	0.441860465	0.761904762
54	PAK4	7	2.184210526	0.000661	0.457831325	0.571428571
55	HDAC3	6	2.210526316	0.002761501	0.452380952	0.666666667
56	CFTR	6	2.210526316	0.007287819	0.452380952	0.266666667
57	PDE4D	6	2.210526316	0.025879498	0.452380952	0.333333333
58	FNTA	5	2.394736842	0.000934	0.417582418	0.5
59	ABCC1	5	2.460526316	0.0000501	0.406417112	0.9
60	PDE10A	5	2.460526316	0.030492877	0.406417112	0.3
61	TGM2	5	2.263157895	0.000547	0.441860465	0.7
62	HDAC11	5	2.618421053	0.001518229	0.381909548	0.7
63	MYLK	5	2.236842105	0.000134	0.447058824	0.9
64	ADORA1	4	2.289473684	0.001456925	0.436781609	0.5
65	TTK	4	2.618421053	0	0.381909548	1
66	F10	4	2.776315789	0.000326	0.360189573	0.666666667
67	ADORA2A	3	2.447368421	0.002355568	0.408602151	0.333333333
68	KLKB1	3	2.789473684	0.000501	0.358490566	0.666666667
69	PDE2A	3	3.131578947	0.001756639	0.319327731	0.666666667
70	CCKBR	2	2.421052632	0.026315789	0.413043478	0
71	PFKFB3	2	2.526315789	0	0.395833333	1
72	DHODH	2	3.197368421	0	0.312757202	1
73	HDAC8	2	2.868421053	0	0.348623853	1

74	PDE5A	2	3.434210526	0	0.291187739	1
75	CCKAR	1	3.407894737	0	0.293436293	0
76	DRD4	1	2.447368421	0	0.408602151	0
77	PLA2G7	1	2.657894737	0	0.376237624	0
78	ADRA1A[a]	0	0	0	0	0

[a]= Did not form any interaction
